# Shock about heat shock in cancer

**DOI:** 10.18632/oncotarget.646

**Published:** 2012-08-30

**Authors:** Emmanuel de Billy, Jon Travers, Paul Workman

**Affiliations:** Cancer Research UK Cancer Therapeutics Unit, Division of Cancer Therapeutics, The Institute of Cancer Research, London, UK; Cancer Research UK Cancer Therapeutics Unit, Division of Cancer Therapeutics, The Institute of Cancer Research, London, UK; Cancer Research UK Cancer Therapeutics Unit, Division of Cancer Therapeutics, The Institute of Cancer Research, London, UK

The transcription factor heat shock factor 1 (HSF1) is the master regulator of the heat shock response. It is crucial for cell homeostasis and implicated in aging, neurodegenerative disease and cancer [1]. Although induction by HSF1 of the expression of molecular chaperones and other regulators of protein quality control, both folding and degradation, is well established, the precise and detailed transcriptional network that HSF1 regulates in cancer is poorly understood. An important new study identifies an HSF1-regulated transcriptional program in highly malignant cells that is surprisingly distinct from the traditional heat shock response [2]. The results have significant implications for our molecular understanding of cancer and the development of new therapies.

The heat shock response, mediated by activation of HSF1, is an ancient, highly conserved mechanism that protects organisms against various adverse environmental and pathological conditions that damage cellular proteins [3]. In cancer, such proteotoxic stress is caused by accumulation of mutated proteins, aneuploidy, reactive oxygen species and the challenging tumor microenvironment (eg hypoxia, low pH), which malignant cells must counteract by activating HSF1 to survive [4, 5].

**Figure 1:**
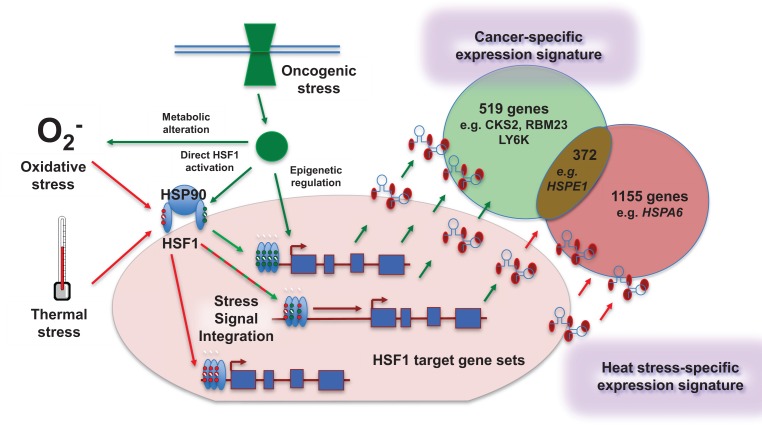
Distinct and overlapping genes are regulated by activation of the heat shock response when induced by cancer versus other cell stresses Mendillo *et al* [2] show that constitutive activation of HSF1 in cancer cells leads to specific binding at promoter and distal sites, resulting in alteration of expression of genes that are distinct from (as well as those overlapping with) the canonical heat shock genes with which the transcription factor is classically associated. Oncogenic stress likely contributes to selection of target genes by HSF1 via several non-mutually exclusive mechanisms. These include changes at the epigenetic level through which HSF1 binding sites are made accessible in cancer cells, together with combinations of different post-translational modifications (depicted by small red and green circles on HSF1) from specific stress-related pathways activated in malignancy. These signals are likely integrated with each other to allow differential gene selection depending on the nature of the stress. The mechanisms provide interesting scope for pharmacological intervention to direct HSF1 activity specifically towards a non-malignant transcriptional program, thereby increasing anticancer activity and therapeutic index.

In healthy, non-stressed cells, HSF1 is located in the cytoplasm in an inhibitory complex with the molecular chaperone heat shock protein 90 (HSP90) – another key player in proteostasis and malignant progression. Upon proteotoxic stress as classically induced by heat shock, HSF1 dissociates from HSP90, trimerizes, translocates to the nucleus and associates with its cognate DNA sequence localized at the promoters of its target genes to modulate their transcription (Figure 1). HSF1 activation by proteotoxic stress is tightly regulated by many post-translational modifications, including phosphorylation, sumoylation and acetylation, the pattern and extent of which varies depending on the nature and the intensity of the stress [3].

Deregulated proteostasis in cancer is a vulnerability, exploitable by targeting protein folding and degradation. The proteasome inhibitor bortezomib (approved in multiple myeloma) and HSP90 inhibitors (showing promise in breast and non small cell lung cancer) enhance proteotoxic stress in cancer cells, causing cell cycle arrest and apoptosis. However, their efficacy is limited by activation of HSF1 and hence induction of numerous cytoprotective proteins, including chaperones like HSP90 and HSP70 family members that ameliorate the proteostatic damage, causing drug resistance [6-8].

Accumulating evidence shows that HSF1 is critically involved in oncogenesis. Key studies using HSF1-knockout mice demonstrate the requirement for HSF1 in tumorigenesis by oncogenic RAS or mutant P53 [9]. Furthermore, HSF1 silencing blocks proliferation and survival of cancer cells driven by diverse oncogenic factors. HSF1 supports the malignant phenotype by promoting oncogenic signal transduction pathways, proliferation, survival, protein synthesis and glucose metabolism [9]. Thus HSF1 is not only a critical factor in drug resistance and regulation of proteotoxic stress, but also a crucial driver in cancer and a potential therapeutic target [10, 11].

The common view is that the key effects of HSF1 in oncogenesis are mediated via increased HSP expression with oncogenic-support properties [10]. However, Medillo *et al.* now demonstrate that HSF1 rewires the transcriptome in cancer in a surprising new way – one that is quite distinct from the classical heat shock response.

First, Medillo *et al* demonstrate that HSF1 protein expression is up-regulated constitutively and also activated – as shown by its nuclear localization and its phosphorylation at serine 326 – in oncogene-transformed breast cancer cells that are highly tumorigenic and metastatic, as compared to their less malignant isogenic counterparts. Also, the most aggressive cancer cell lines are more dependent on HSF1 for their growth and survival, indicating a stronger ‘addiction’ and therapeutic vulnerability.

Next, Medillo *et al.* use chromatin immunoprecipitation linked to next-generation sequencing (ChIP-Seq) to identify the genes bound by HSF1. Under basal conditions HSF1 binds many more genes in the highly malignant compared to less aggressive cancer cells or immortalized but non-transformed cells. Binding occurs at both promoter regions and distal sites. Following heat shock, HSF1 binding to genes is enhanced in all the cell lines. Surprisingly, however, there are marked differences in the genes bound by HSF1 in cancer under basal conditions versus normal cells following heat shock. Many genes bound specifically in the aggressive breast cancer cells are not classical heat shock proteins and there is enrichment for genes involved in protein translation, RNA binding, metabolism and adhesion. Examples of cancer-specific genes highlighted by Medillo *et al*. include *CKS2* encoding cyclin-dependent kinase interacting protein; *LY6K* coding for glycophosphatidyl-inositol-anchored membrane protein with cancer connections; and *RBM23* encoding an RNA-binding protein implicated in estrogen-mediated transcription.

On the other hand, another set of genes bound by HSF1 in the cancer cell lines under basal conditions are common to those binding in non-transformed cell lines after heat shock. Thus the results indicate both interesting differences and a level of overlap between the HSF1 gene signature regulated by thermal stress and that associated with malignancy – raising the possibility that different molecular mechanisms (eg epigenetic regulation and various post-translational modifications) may be preferentially involved under these two stress-activating conditions (Figure 1). Importantly, Medillo *et al*. use RNA interference to confirm the role of HSF1 in regulating transcription of its identified bound genes.

Following on, Medillo *et al.* confirm the identified cancer-specific HSF1 gene occupancy profile in cancer cells from diverse tumor origins including breast, colon and lung. Of high translational significance, they then use ChIP-Seq to confirm that the cancer-specific HSF1-mediated gene transcription program is active in breast, colon and lung tumors obtained directly from human patients.

Finally and of direct clinical relevance, Medillo *et al.* demonstrate that an ‘HSF1-cancer gene signature’ comprising 456 cancer HSF1-regulated genes – which include the cancer-specific set and the cancer-regulated genes overlapping with the heat shock set – is associated with poor clinical outcome in diverse human cancers including breast, colon and lung cancer and is more predictive than other commonly used prognostic markers such as MYC, Ki67 and the MammaPRINT gene signature.

This important new study provides a much clearer understanding of the role of HSF1 in progression to the highly malignant and metastatic state – as well as much food for thought for future research. Most significant and surprising is the specific rewiring of the transcriptome by HSF1 in cancer cells. The new work also highlights the complexity of the mechanisms implicated in HSF1 regulation and function. Differences in the genes bound by HSF1 between the highly aggressive versus less malignant cell lines could reflect disparities in the amount of active HSF1 present in the nucleus or may result from the deregulation of several key oncogenic pathways (eg EGFR/HER2, RAS/MAPK and insulin/IGF1) and/or epigenetic states.

In addition to the implications for basic research there are important translational consequences. Understanding the functional relevance of the epigenetic factors and post-translational modifications that modulate HSF1 activity, as well as identifying the proteins involved and the cofactors associated with HSF1 transcriptional activity, is likely to provide both new cancer biomarkers and novel ways to target more specifically and efficiently (eg Figure 1) the megalomaniac role played by HSF1 in malignancy.

Thus, although HSF1 has been around for a billion years of evolution, it continues to surprise us. In the near future we will very likely see more shocks about heat shock in cancer.
